# Clinical characteristics of skin pigmentation caused by pemetrexed: a case report and literature review

**DOI:** 10.3389/fmed.2025.1697371

**Published:** 2025-11-06

**Authors:** Leling Zhou, Li Chen, Xin Cao, Ying Wang, Ting Jiang

**Affiliations:** 1 Department of Pharmacy, Wusheng People's Hospital, Guangan, Sichuan, China; 2 Department of Oncology, Wusheng People's Hospital, Guangan, Sichuan, China; 3 Department of Pharmacy, Clinical Medical College and The First Affiliated Hospital of Chengdu Medical College, Chengdu, Sichuan, China

**Keywords:** adenocarcinoma of the lungs, skin pigmentation, pemetrexed, adverse drug reaction, chemotherapy

## Abstract

Pemetrexed demonstrates significant efficacy and safety in second-line and single-agent maintenance therapies, and in combination with platinum-based chemotherapy, it serves as the standard first-line treatment for driver-negative advanced non-small cell lung cancer (NSCLC). Although several studies have reported its widespread use and associated adverse effects, reports of pemetrexed-related pigmentation are rare. At present, the mechanism underlying pemetrexed-induced pigmentation remains unknown, and the timing of onset and risk factors are unclear. Here, we report a case of left lung adenocarcinoma (cT2bN2M1 IVB) with bone metastasis. After the third cycle of pemetrexed, pigmentation, numbness, pain, and walking difficulty developed on both feet and the dorsum of the ankles. These symptoms gradually worsened with subsequent chemotherapy cycles. Following neurotrophic treatment with diclofenac sodium gel and mecobalamin, numbness and pain improved, while pigmentation subsided gradually after discontinuation of pemetrexed. In addition, this study retrospectively analyzed nine patients with pemetrexed-induced skin pigmentation and found that the median onset was during the second cycle (range: 1–17 cycles). Pigmentation could be widely distributed on the body surface and aggravated with continued pemetrexed therapy. However, pigmentation resolved spontaneously after discontinuation, with individual differences.

## 1 Introduction

According to the 2022 Global Cancer Statistics Report, lung neoplasms have the highest incidence and mortality rates worldwide (1). Furthermore, the mortality rate of lung cancer has slightly increased since 2020 (2). Patients with early-stage lung neoplasms undergo surgical procedures, such as thoracoscopy, for lesion removal. Although the continuous development of molecularly targeted drugs has brought new hope to patients with driver gene-positive advanced lung neoplasms, chemotherapy remains the primary treatment for those with driver gene-negative disease. Currently, pemetrexed combined with platinum-based chemotherapy is the standard first-line treatment for driver-negative advanced NSCLC and also exhibits significant efficacy and safety in second-line and single-agent maintenance settings (3–8, 43). As a multi-targeted folate antagonist (9), pemetrexed is primarily used for NSCLC and malignant pleural mesothelioma (10, 11). Its common adverse effects include myelosuppression and skin toxicity. However, few national and international studies have examined the adverse reaction of pemetrexed-related skin pigmentation. This report describes a case of pigmentation in an advanced NSCLC patient treated with pemetrexed, reviews the relevant literature, and summarizes the clinical manifestations and management strategies of pemetrexed-induced pigmentation. Additionally, it highlights high-risk groups for this adverse event and explores the potential correlation between pigmentation and treatment efficacy.

## 2 Case description

**Figure 1:**
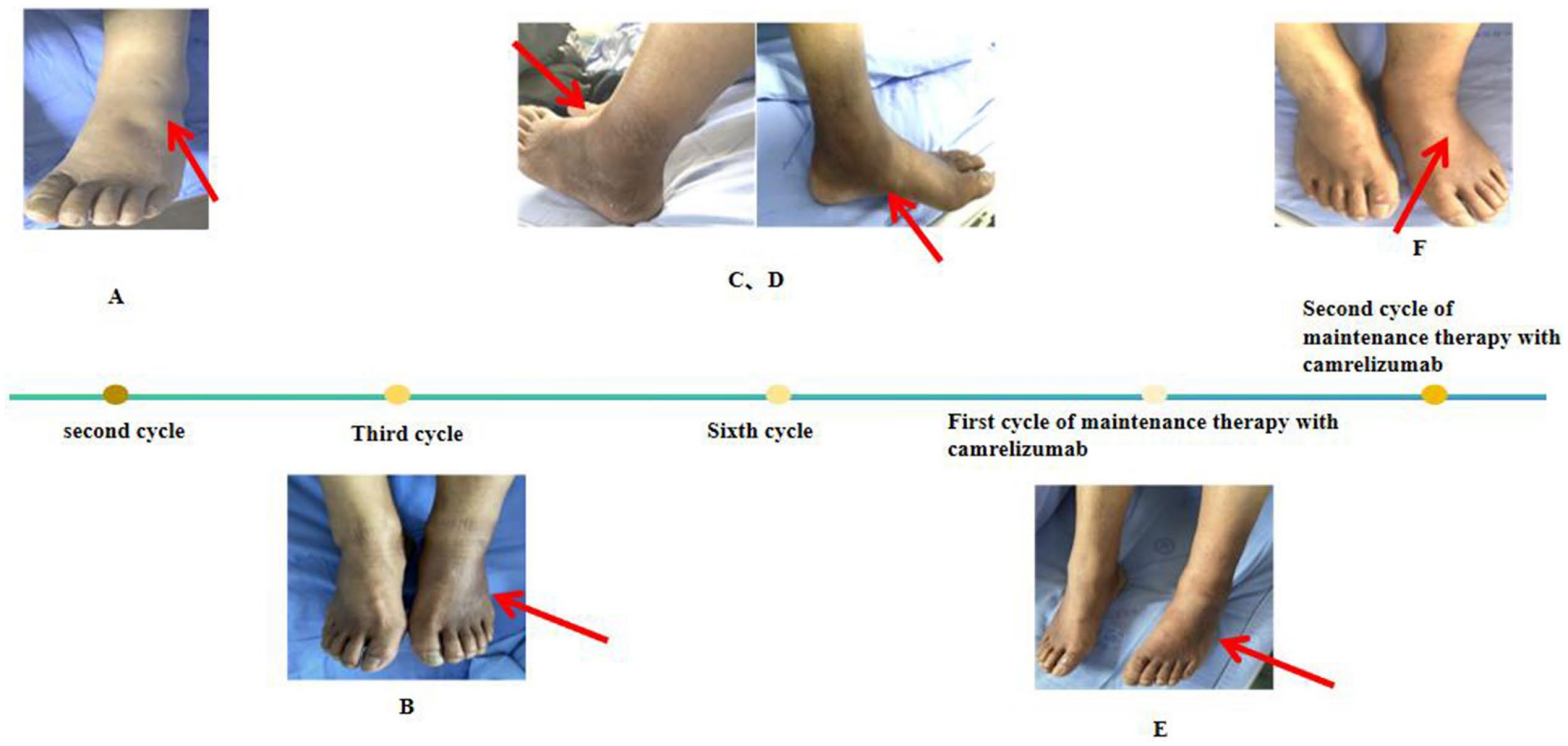
**(A)** The patient experienced numbness in the soles of both feet, along with moderate pitting edema in both lower limbs, more pronounced in the left lower limb, with significant tenderness. The skin showed no pigmentation, ecchymosis, or petechiae, and the skin temperature was not elevated upon palpation. **(B)** The patient still had edema in the anterior tibial region of both lower limbs, with new patchy black pigmentation and desquamation observed on the dorsum of both feet and ankles, more prominent on the dorsum and ankle of the left foot. The numbness and pain were significantly worse than before, with no erosion, blisters, or lichenification observed. **(C,D)** After this chemotherapy, the patient still experienced numbness in the soles of both feet, with increased pigmentation and desquamation accompanied by pain in the left ankle. **(E,F)** The pigmentation in the patient’s lower extremities gradually diminished.

A previously healthy 65-year-old man was diagnosed with left lung adenocarcinoma with bone metastasis (cT2bN2M1 IVB). He had a smoking history of more than 40 years, with a consumption of about 40 cigarettes per day. After diagnosis, the patient received four cycles of camrelizumab (200 mg, ivgtt d0), pemetrexed (700 mg, ivgtt d1), and carboplatin (500 mg, ivgtt d1) from 22 November 2023. During this period, he underwent left hip radiotherapy (4,000 Gy/20 f) on 10 October 2023. Pre-treatment was administered according to chemotherapy guidelines. Following two cycles, the patient developed bilateral plantar numbness, moderate depression, edema in both lower limbs, and severe tenderness in the left lower limb (Figure 1A). In the third cycle, patchy black pigmentation appeared on the heels and ankles of both feet (Figure 1B), which worsened with subsequent chemotherapy cycles. After the fourth cycle, due to carboplatin-related grade IV myelosuppression (hemoglobin: 57 g/L), carboplatin was discontinued from the fifth cycle onward, and the patient continued to receive camrelizumab (200 mg, ivgtt d0) and pemetrexed (600 mg, ivgtt d1).

The patient remained capable of self-care and minor physical activities, such as walking and simple household tasks. He experienced mild lower limb pain that did not affect sleep. Therefore he showed Performance Status (PS) and Numeric Rating Scale (NRS) scores of 1 and 2, respectively. Vital signs were stable, and he was conscious and responsive. Significant pigmentation was observed on both lower limbs, particularly on the dorsum of the left foot and ankle (Figures 1A,B). Laboratory investigations, including complete blood count and liver and renal function tests, were normal. Chest computed tomography (CT) findings indicated treatment efficacy evaluated as Partial Response (PR). With no apparent contraindications to chemotherapy, the patient underwent a sixth cycle of camrelizumab (200 mg, ivgtt d0) and pemetrexed (600 mg, ivgtt d1) on 29 March 2024. However, he continued to experience plantar numbness, with increased pigmentation, desquamation, and pain in the left ankle (Figures 1C,D). Diclofenac sodium gel was applied topically three times daily to alleviate pain, and mecobalamin (one tablet) was administered orally three times daily to support nerve health. Subsequently, pemetrexed was discontinued, while camrelizumab was continued for two cycles as maintenance therapy. During this period, pigmentation in the lower extremities gradually decreased (Figures 1E,F).

## 3 Discussion

According to Naranjo’s scale for assessing adverse drug reactions, the correlation between pemetrexed and pigmentation was analyzed. After the second cycle, the patient developed edema in both lower extremities and bilateral plantar numbness, followed by progressive black pigmentation of the lower extremities from the third to sixth cycles. Numbness and pain worsened over time, leading to walking difficulty and hyperpigmentation. The use of pemetrexed has been associated with pigmentation in some patients. After six cycles, the treatment efficacy was evaluated as Partial Response (PR). Thereafter, camrelizumab monotherapy was continued as maintenance therapy. During this period, pigmentation of the lower limbs gradually subsided. According to the prescribing information for pemetrexed, carboplatin, and camrelizumab, as well as clinical trial data, the most common adverse reactions of pemetrexed are myelosuppression, rash, and occasional pigmentation (3–8, 12–14). Neither carboplatin nor camrelizumab has been reported to cause pigmentation. Peripheral neurotoxicity is a known adverse effect of platinum drugs, often manifesting as numbness of the hands and feet. Compared with cisplatin and oxaliplatin, carboplatin has minimal neurotoxicity, occurring in only 4%–6% of patients, typically after high-dose administration (15–17). In this patient, peripheral neurotoxicity did not improve after carboplatin was discontinued following the fourth cycle due to anemia. However, after discontinuation of pemetrexed, the patient’s lower limb numbness was significantly reduced, suggesting that carboplatin was unlikely to be the cause of pigmentation or neuropathy. Thus, the pigmentation was considered unrelated to carboplatin and camrelizumab.

Further investigations, such as lower extremity vascular ultrasound, thyroid function, adrenocorticotropic hormone (ACTH), and antinuclear antibody tests, are useful to exclude rheumatic or immune-related diseases. Lung adenocarcinoma can also cause paraneoplastic syndromes, including pigmentation (18), which generally resolves when the underlying condition improves. In this case, however, pigmentation worsened despite clinical improvement of the malignancy. Based on this analysis, and with a final Naranjo’s scale score of 7 (a score of ≥9 indicates definite, 5–8 probable, 1–4 possible, and ≤ 0 doubtful), the patient’s pigmentation was judged as probably caused by pemetrexed. According to the Common Terminology Criteria for Adverse Events (CTCAE v5.0), pigmentation involving <10% of the body surface area is classified as grade 1. Table 1 presents Naranjo’s probability scale for assessing pemetrexed-associated pigmentation.

**Table 1:** Naranjo’s probability scale for assessing pemetrexed-induced pigmentation.

To assess the adverse drug reaction, please answer the following questionnaire and give the pertinent score.				
	Yes	No	Do not know	Score
1. Are there previous conclusive reports on this reaction?	+1	0	0	+1
2. Did the adverse event appear after the suspected drug was administered?	+2	−1	0	+2
3. Did the adverse reaction improve when the drug was discontinued or a specific antagonist was administered?	+1	0	0	+1
4. Did the adverse reaction reappear when the drug was readministered?	+2	−1	0	0
5. Are there alternative causes (other than the drug) that could, on their own, have caused the reaction?	-1	+2	0	+2
6. Did the reaction reappear when a placebo was given?	-1	+1	0	0
7. Was the drug detected in the blood (or other fluids) in concentrations known to be toxic?	+1	0	0	0
8. Was the reaction more severe when the dose was increased, or less severe when the dose was decreased?	+1	0	0	0
9. Did the patient have a similar reaction to the same or similar drugs in any previous exposure?	+1	0	0	0
10. Was the adverse event confirmed by any objective evidence?	+1	0	0	+1
			Total score	7

Extensive clinical trial data indicate that pemetrexed is a widely used cytotoxic chemotherapy drug with cell cycle specificity for advanced NSCLC and demonstrates good efficacy in advanced breast neoplasms as well (19, 20). Pemetrexed, a multi-targeted antifolate antagonist, exerts tumor-specific activity (9). Calvert (21) showed that pemetrexed acts on multiple enzymes, reducing tetrahydrofolate synthesis through dihydrofolate reductase inhibition, thereby impairing thymidine nucleotide synthesis. It also inhibits glycinamide ribonucleotide formyltransferase (22). Hwang et al. (23) further demonstrated that pemetrexed targets aminoimidazole carboxamide ribonucleotide formyltransferase. Pigmentation induced by other antimetabolite cytotoxic agents, including 5-fluorouracil (24), hydroxyurea (25), and capecitabine (26), has been frequently reported. Although the distribution and morphology of pigmentation differ among drugs, most cases are reversible and subside upon drug discontinuation. While the adverse effects of pemetrexed are well documented, only a few reports have described pigmentation.

**Figure 2:**
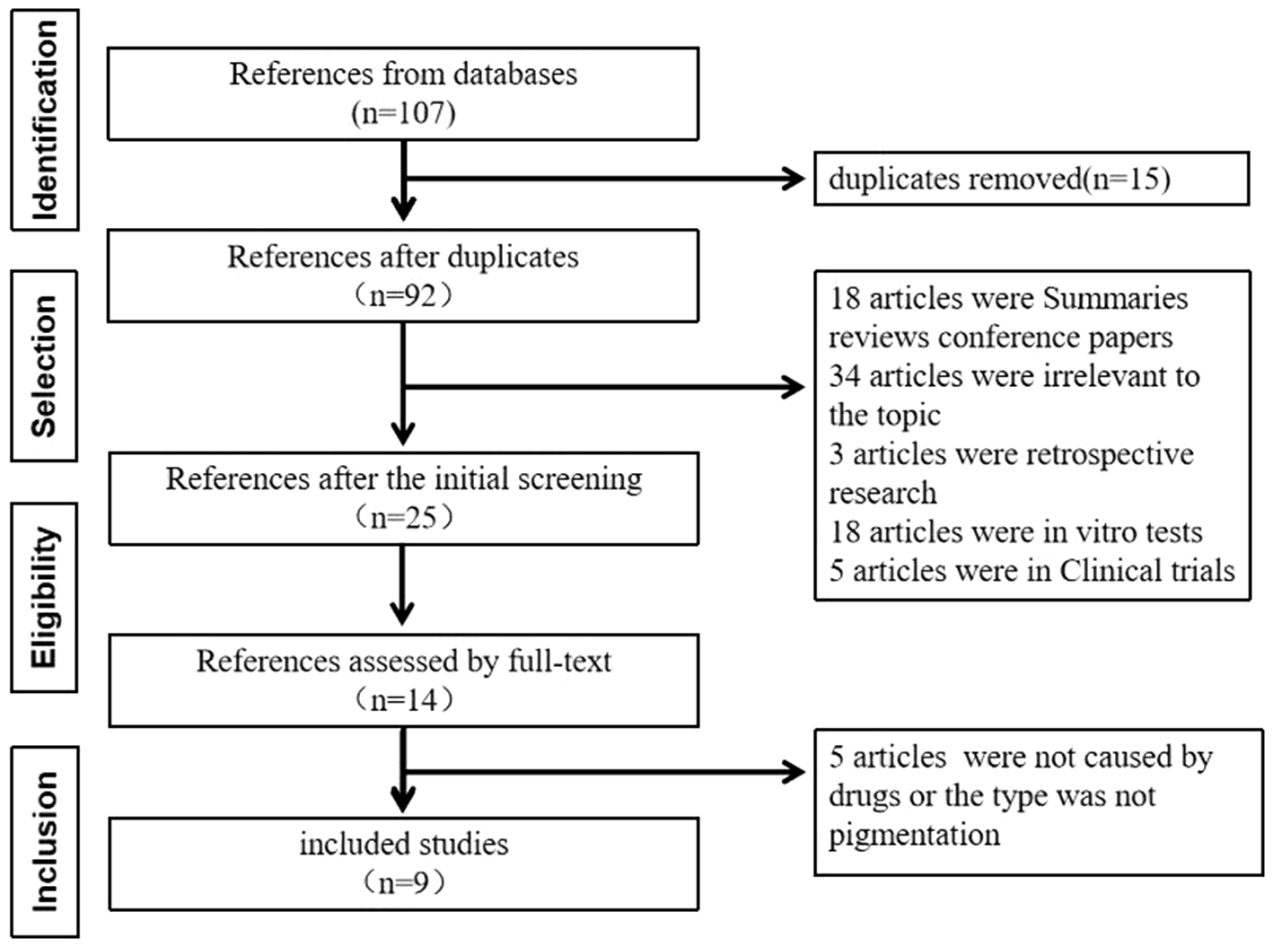
The schematic of the literature screening.

A literature search was performed following the PRISMA statement guidelines. Case reports of pemetrexed-induced pigmentation published in PubMed, EMBASE, CNKI, China Wanfang, and China VIP databases up to 31 December 2024 were reviewed. Both free-text and subject-heading searches were conducted using the terms “Pemetrexed Disodium,” “Pemetrexed,” and “Pigmentation.” A total of 107 articles were identified, including 61 from PubMed, four from EMBASE, nine from CNKI, 14 from China Wanfang, and 19 from China VIP. After excluding 98 publications such as summaries, duplicates, reviews, unrelated topics, retrospective studies, *in vitro* experiments, and conference papers, nine articles were finally included. A schematic of the literature screening is shown in Figure 2. The summary of published case reports on pemetrexed-induced pigmentation is presented in Table 2.

**Table 2:** Summary of all published case reports on pemetrexed-induced pigmentation.

Author	Country	Year	Patient/cancer type	Chemotherapy regimen	Occurrence cycle	Clinical manifestation	Treatment measures	Prognosis
Zhao et al. (27)	China	2013	62/M/Lung bronchoalveolar carcinoma	Pemetrexed	1	The forehead, eyelids, cheekbones, palms, palm prints, and soles of the feet has noticeably darkened, accompanied by pigmentation.	VC 5.0 g/qd + reduced glutathione1.8 g/qd q21d	After two cycles, the pigmentation gradually fades, and after four cycles, the skin returns to its pre-chemotherapy state.
Gong et al. (28)	China	2017	60/F/NSCLC (adenocarcinoma EGFR19-Del mutation)	Pemetrexed+Cisplatin	2	The skin on the forehead, around the eyelids, forearms, back of the palm, back of the foot, and toes visibly darkened, accompanied by pigmentation.	Untreated	When followed up 1 month after the completion of four cycles of chemotherapy, the pigmentation had basically disappeared, and after 2 months, the pigmentation had completely disappeared.
Yuan et al. (29)	China	2015	52/M/NSCLC(adenocarcinoma)	Pemetrexed+Cisplatin	2	Extensive pigmentation in the axillary region.	untreated	Gradual disappearance after discontinuation of pemetrexed
Wang et al. (30)	China	2016	58/M/NSCLC (adenocarcinoma)	Pemetrexed+Cisplatin	1	The range of pigmentation gradually changes from the face to the whole body, especially in the skin folds of the neck, armpits, surgical incisions, waist, palms, and soles.	Untreated	Gradual disappearance after discontinuation of pemetrexed
Wu et al. (31)	China	2017	64/M/Lung cancer	Pemetrexed+Carboplatin	2	Mild skin darkening with desquamation was observed, which gradually aggravated, and the skin with systemic pigmentation showed similar desquamation changes.	Untreated	After 14 days of discontinuation, the hyperpigmented skin has completely shed, and the skin has returned to normal.
Buchinger et al. (32)	Switzerland	2013	77/M/Malignant pleural mesothelioma	Pemetrexed+Carboplatin	17	The skin of the whole body is pigmented except for the soles of both hands and feet.	Untreated	Not described in the text
Schallier et al. (33)	Belgium	2011	59/M/NSCLC(adenocarcinoma)	Pemetrexed	2	A ‘brownish’ hyperpigmentation of both palms of the hands and soles of the feet. The skin felt dry and raspy.	Untreated	After discontinuing pemetrexed for three weeks, it completely disappeared due to disease progression and eventual death.
Dasanu et al. (34)	America	2007	53/M/NSCLC(adenocarcinoma)	Pemetrexed	3	Transverse and longitudinal black lines appeared on his fingernails and toenails. And diffuse black discoloration appeared in the nails at the late stage of chemotherapy.	Untreated	Not described in the text
Vashisht et al. (35)	India	2018	57/F/NSCLC	Pemetrexed+Cisplatin	6	Multiple asymptomatic, well-defined, and irregular linear pigmentation spots appear on the distal limbs, except for the palms and soles of the feet.	Untreated	Death due to neutropenic sepsis
Zhou et al. (this paper)	China	2024	65/M/NSCLC (adenocarcinoma)	Pemetrexed+Cisplatin	3	The lower limbs were initially edematous, and bilateral plantar numbness. Post-third pemetrexed cycle, pigmentation, numbness, pain, and walking disorder appeared on both feet and the ankle dorsum.	Diclofenac sodium gel was applied topically thrice a day to alleviate pain, and mecobalamin (one tablet) was administered orally, thrice a day, to nourish the nerves.	After stopping pemetrexed for two cycles, pigmentation gradually subsided.

In total, 10 patients were analyzed (27–35), including six from China and one each from Switzerland, Belgium, the United States, and India. Nine patients had lung cancer, and 1 had malignant pleural mesothelioma. In terms of chemotherapy regimens, seven patients received pemetrexed combined with platinum, while three were treated with pemetrexed monotherapy. Pigmentation was observed in various body sites, including the forehead, periorbital region, face, limbs, and axillae, with nails being a particularly site. The chemotherapy regimens of ten patients suggest that pemetrexed with platinum drugs causes extensive pigmentation compared to pemetrexed alone. But after using SPSS 27.0 version, no significant differences were observed between the two as per Fisher’s exact test. The relationship between the treatment regimen containing pemetrexed and the extent of pigmentation is detailed in Table 3.

**Table 3:** Pemetrexed treatment regimen and pigmentation range.

Chemotherapy regimen	Total, *n*	Wide range of pigmentation, *n*(%)	Limited pigmentation range, *n*(%)	*P*
Pemetrexed	3	1(33.3)	2(66.7)	0.5
Pemetrexed combined with platinum	7	5(71.4)	2(28.6)	

The median onset of pigmentation was during the second cycle (range: 1–17 cycles) (27–35). Most cases were asymptomatic; only two patients experienced pigmentation accompanied by pain, and none developed itching. Among the ten included patients, only two patients underwent the treatment; the rest were kept for observation, and the dose of pemetrexed was not adjusted. Three patients did not report outcomes, one died due to sepsis, and the pigmentation of the remaining six patients was reversible. Pemetrexed generally subsided after drug discontinuation, although the regression time varied among individuals. The patient in this report developed pigmentation after the third cycle, which became more pronounced with subsequent cycles. Pigmentation was primarily concentrated in the lower limbs and accompanied by numbness, pain, and walking difficulties. Diclofenac sodium gel was used to alleviate pain, and mecobalamin tablets were administered to nourish the nerves. The pigmentation gradually subsided after discontinuation of pemetrexed.

The mechanism of antitumor drug-induced pigmentation is not fully understood, but several explanations have been proposed (36). These include stimulation of melanocytes to produce excessive melanin, accumulation of melanin-drug complexes in skin macrophages that cannot be cleared promptly, adrenal toxicity leading to decreased adrenocorticotropic hormone secretion and increased melanocyte-stimulating hormone secretion, and keratin toxicity causing post-inflammatory pigmentation. Additionally, ultraviolet (UV) irradiation may contribute significantly. The mechanism of pemetrexed-induced pigmentation remains unclear. Because edema of the lower limbs preceded pigmentation in this patient, it is hypothesized that keratin toxicity might be involved. Although pigmentation generally does not interfere with daily activities, patients with facial pigmentation may refuse pemetrexed because of cosmetic concerns. Management options for keratin-related pigmentation include vitamin C with reduced glutathione (27), quinone, azelaic acid, retinoic acid, or vitamin C for local medication (37), and may also benefit from laser treatments (38).

However, due to the complexity of pigmentation mechanisms, the effectiveness of these treatments varies, and standardized protocols are lacking. Whether traditional Chinese medicine can improve pigmentation requires further clinical evidence (39). Preventive measures are recommended, including sun protection with umbrellas, hats, and sunglasses, avoidance of prolonged outdoor activities during midday, and apply broad-spectrum sunscreen before going out to reduce UV exposure and lower the risk of pigmentation.

According to global clinical trial data, pemetrexed is widely used as maintenance therapy for NSCLC (13). Among 438 patients treated with pemetrexed, nine developed pigmentations, with an incidence of 0.91%. In contrast, the incidence in the Chinese population was higher, at 9.7% (9/67). Zhang et al. (40) investigated the relationship between pemetrexed-induced pigmentation and therapeutic efficacy, reporting a pigmentation incidence of 43.6% (51/117), far exceeding that listed in the drug manual. Moreover, patients who developed pigmentation showed higher treatment efficacy, suggesting a possible correlation that warrants further study. Different clinical trials (3–8, 12, 13) involved diverse ethnic groups, and the association between pigmentation and genetic predisposition remains unclear in real-world populations. Several studies have also noted that pemetrexed administered after radiotherapy can cause pigmentation at the irradiated site (41, 42). In this report, three patients had received radiotherapy: two before and one after pemetrexed administration (29, 33). Although pigmentation did not occur at the radiotherapy site in these cases, further studies are needed to clarify whether radiotherapy is a risk factor for pemetrexed-related pigmentation.

## 4 Conclusion

An advanced lung adenocarcinoma patient developed pigmentation with numbness, pain, and walking difficulty on the dorsum of both feet and ankles after pemetrexed treatment. Following therapy with diclofenac sodium gel and mecobalamin, the pigmentation gradually resolved after discontinuation of pemetrexed. Clinical pharmacists reviewed the relevant literature, which revealed that only a few reports have described pemetrexed-induced pigmentation and that no standardized treatment regimen exists. This report summarizes the potential mechanisms, clinical manifestations, and management strategies of pemetrexed-related pigmentation, providing useful references for clinicians and exploring its association with susceptible populations and treatment efficacy. Further research with larger sample sizes is required.

## Data Availability

The datasets presented in this study can be found in online repositories. The names of the repository/repositories and accession number(s) can be found in the article/supplementary material.
